# Three-Dimensional Reconstruction of the Equine Palmar Metacarpal Region Using E12 Plastinated Sections

**DOI:** 10.3390/ani16030449

**Published:** 2026-02-01

**Authors:** Gulsum Eren, Octavio López-Albors, Mirian López Corbalán, Rafael Latorre

**Affiliations:** Department of Anatomy and Comparative Pathological Anatomy, Veterinary Faculty, University of Murcia, 30100 Murcia, Spain; miriam.lopez15@um.es (M.L.C.); latorre@um.es (R.L.)

**Keywords:** veterinary anatomy education, horse metacarpal region, E12 sheet plastination, three-dimensional modeling, 3D reconstruction

## Abstract

Digital technologies have improved the visualization of anatomical structures for veterinary education and clinical practice. In this study, a detailed three-dimensional anatomical model of the equine palmar metacarpal region was generated using E12-based epoxy sheet plastination combined with digital reconstruction in Amira^®^ V5.6 software. Serial cross-sections of the metacarpal region provided high-resolution visualization of bones, tendons, ligaments, nerves, vessels, fasciae, and synovial structures, with minimal shrinkage or deformation, ensuring improved anatomical accuracy. These sections were digitized, aligned, and manually segmented to accurately delineate anatomical boundaries, particularly in areas of low contrast. The resulting three-dimensional model represents the topographical relationships of key structures, including palmar nerves and vessels, the palmar fascia with the metacarpal flexor retinaculum (MFR), and the common synovial sheath (*Vag. synovialis communis mm. flexorum*, CSS). The model allows rotation and selective visualization of individual structures, facilitating examination from multiple perspectives. This combined plastination–digital approach provides an accurate anatomical reference with value for veterinary anatomy education, clinical training, surgical planning, and research on equine musculoskeletal disorders.

## Simple Summary

Understanding the topographic locations and relationships of anatomical structures is essential for anatomy education. Technology-supported visual resources provide valuable references for both students and clinicians. This study represents a valuable contribution, as the equine metacarpal region was modeled using plastinated sections and Amira^®^ V5.6 software, providing a detailed three-dimensional anatomical reference. The 3D model clearly illustrates the positions and connections of bones, tendons, ligaments, vessels, nerves, and connective tissues. Users can rotate and examine the model from different angles, which enhances understanding of the spatial relationships among structures. By combining plastinated sections with digital tools, this study provides a reliable and practical reference for anatomy education, clinical practice, and research on equine musculoskeletal anatomy.

## 1. Introduction

Books, illustrations, and digital materials are important in anatomy education and research, but cadavers—preserved through methods such as plastination—remain invaluable, providing long-lasting, realistic specimens that maintain their original morphology and thereby are suitable for learning and research [1]. E12 epoxy sheet plastination is particularly effective for detailed mesoscopic examination compared to conventional preservation methods. It preserves tissue arrangement with minimal shrinkage, providing clear topographic and anatomical details. The transparency of connective tissues also allows easy identification of fibrous structures [2].

Digital technologies play an increasingly important role in anatomical visualization, teaching strategies, and clinical preparation. Consequently, anatomy education and veterinary practice are progressively supported by species- and patient-specific digital models, facilitating preoperative planning and minimizing surgical risks [3,4]. Clinical students may improve their manual and technical skills through virtual surgical simulations before performing real procedures, and models with realistic clinical scenarios may also enhance their decision-making and problem-solving abilities [3,4,5,6]. In addition, plastinated anatomical specimens and 3D digital models provide highly accessible anatomical resources that support both individual study and remote learning environments, including online teaching and interdisciplinary meetings. Digital 3D models and plastinated samples can overcome spatial limitations, e.g., by enabling virtual anatomy museums that reach broader audiences, while their interactivity and high visual quality provide substantial educational, scientific, and cultural value [7,8].

Musculoskeletal disorders are common in sport horses, often causing long recovery or career-ending problems. Lesions in the proximal and palmar metacarpal regions are clinically significant and are therefore examined using various imaging methods [9,10]. Alongside these diagnostic and therapeutic advancements, newly developed treatment protocols, regenerative medicine approaches, and surgical techniques continue to diversify clinical applications in this field [4,11,12,13]. However, despite these improvements, high-quality three-dimensional anatomical models that accurately represent bones, tendons, vessels, nerves, and delicate connective tissues, such as fascia and synovial sheaths, remain lacking. The E12 epoxy sheet plastination technique preserves these tissues with minimal shrinkage or deformation, providing clear visualization of their topography and spatial relationships, and offering a unique and indispensable tool for both anatomical education and precise clinical planning [2].

Therefore, the aim of this study was to develop a three-dimensional anatomical model that accurately represents the musculoskeletal and neurovascular structures of the metacarpal region of a horse, including the palmar fascia with the metacarpal flexor retinaculum (MFR) and the common synovial sheath (*Vag. synovialis communis mm. flexorum*, CSS), using cross-sections from the sheet plastination technique. This model provides a clear and precise tool for teaching veterinary anatomy and for understanding and addressing orthopedic problems.

## 2. Materials and Methods

The right forelimb of a two-year-old mare was obtained from a local abattoir. The animal had no recorded locomotor disorders and had not undergone any specialized athletic training, as it had been raised as a farm animal. A single specimen (*n* = 1) was used, which is considered sufficient for detailed anatomical reconstruction studies. The distal limb was transported to the dissection room at the Faculty of Veterinary Medicine at the University of Murcia within a maximum of two hours post-slaughter. Upon arrival, the limb was thoroughly washed, and the joints were realigned into their anatomical positions, with special attention given to the metacarpophalangeal joint. This joint was positioned by attaching a wire from the dorsal side of the third metacarpal bone to the midpoint of the dorsal edge of the solear border of the hoof. The limb was then immediately frozen at −20 °C for seven days to ensure thorough freezing of the tissues, which helps preserve their natural anatomical structure and minimize distortion during subsequent processing.

### 2.1. Cryosectioning

One week after initial freezing, the limb (*n* = 1) was released from the wires used to maintain correct joint positioning and transferred to a deep freezer (−76 °C), where it was stored for 10 days. The sample was obtained from the limb with a high-speed band saw from 3 cm distal from the proximal border of the metacarpal bone III to 10 cm proximal to the proximal sesamoid bones.

### 2.2. Plastination

**Figure 1:**
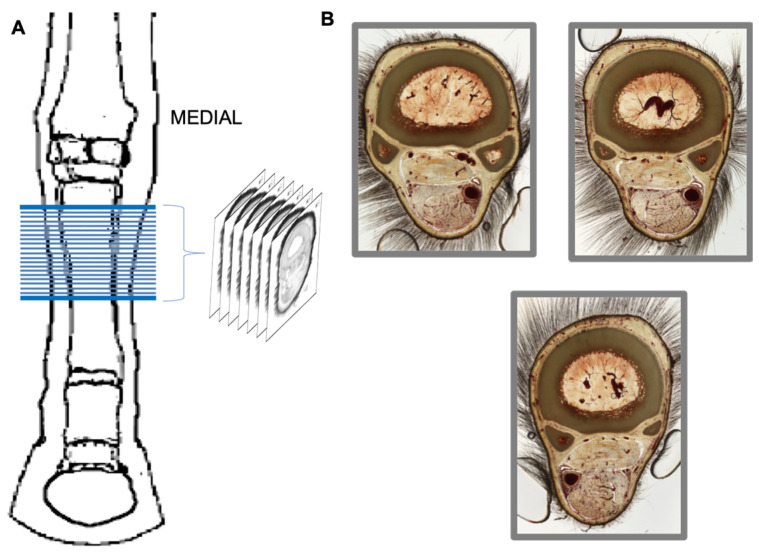
Dorsal view of the distal limb from which the plastinated sections were obtained (**A**), and plastinated section samples from the metacarpal region (**B**).

The sample from the metacarpal region for plastination was serially cross-sectioned with a high-speed band saw (Biodur^®^ Products GmbH, Heidelberg, Germany). The sections were immediately immersed in cooled acetone (−10 °C), and the surfaces of the slices were gently cleaned with a brush. A total of 29 sections were obtained (Figure 1). Sections were transferred to a bath of cooled pure acetone (−15 °C) for further E12 plastination processing (impregnation and curing), as described by Sora et al. [1]. At the end of the procedure, 29 epoxy-plastinated sections were obtained (Figure 1).

### 2.3. Digitization of the Images

Epoxy plastinated sections were scanned using an Epson Expression 1680 Pro scanner (Seiko Epson Corporation, Suwa, Nagano, Japan) to convert the physical specimens into high-resolution digital images. Due to the 2 mm thickness of the sections, both proximal and distal surfaces were scanned separately to avoid information loss. A resolution of 600 dpi, 24-bit color depth, and 1990-pixel width was applied to ensure proper alignment, and the images were saved in TIFF file format.

### 2.4. Three-Dimensional Model Reconstruction Using Amira Software

**Figure 2:**
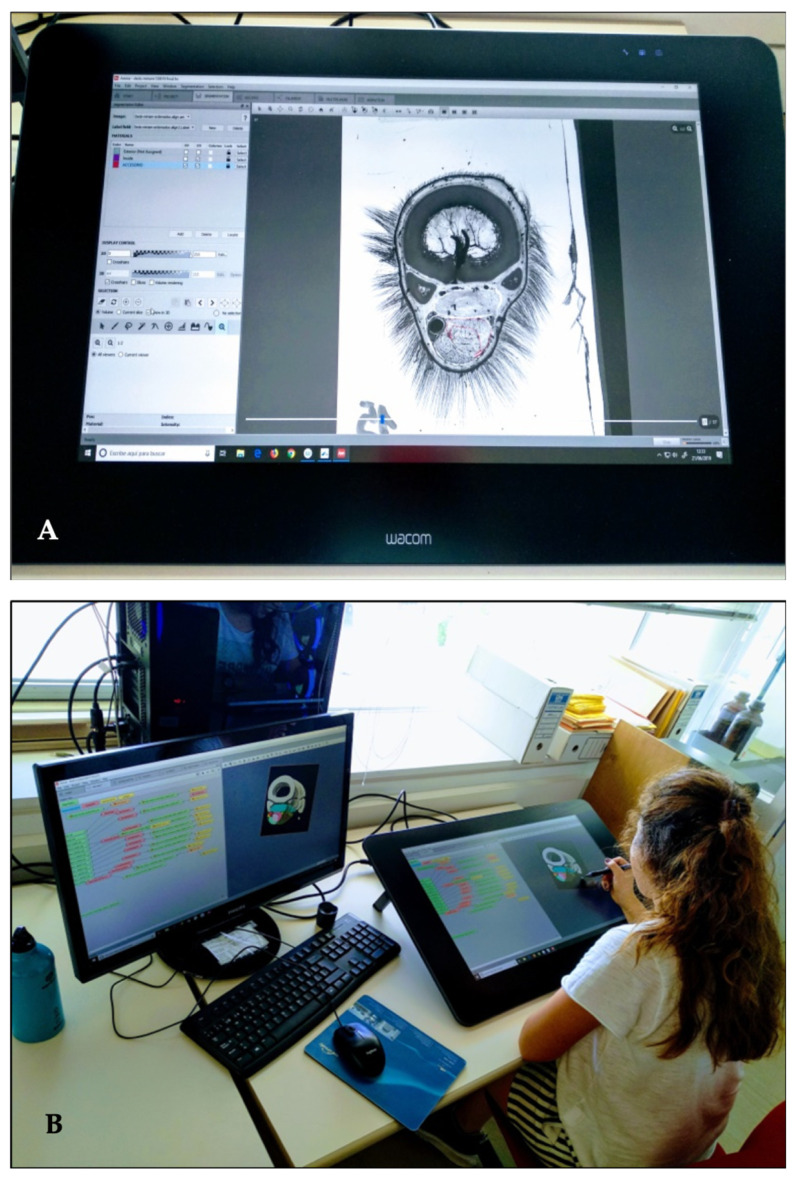
Outlining of anatomical structures in plastinated scanned sections process. (**A**). Raw scanned image used as input for Amira processing. (**B**). Segmented silhouettes of anatomical structures on each scanned section, rendered in Amira.

The scanned plastinated cross-sections, saved as high-resolution TIFF image stacks, were imported into Amira^®^ V5.6 (Thermo Fisher Scientific Inc., Waltham, MA, USA). A standardized workflow was applied for image processing, alignment, segmentation, and three-dimensional reconstruction (Figure 2A). First, slice-to-slice alignment was performed using rigid transformations (translation and rotation) to ensure the correct spatial orientation of the section series. Automatic alignment was complemented with manual adjustments to correct minor variations in slice geometry and low contrast of some soft tissue structures.

Following alignment, segmentation of anatomical structures was carried out using a combination of manual tracing tools, threshold-guided selection, and region-based refinement (Figure 2B). Manual delineation was preferred for soft tissue borders, including fasciae, ligaments, tendons, and neurovascular structures, because automatic segmentation was insufficient to accurately distinguish interfaces where contrast gradients were weak. This approach ensured precise boundary identification of the complex anatomical structures characteristic of the palmar metacarpal region, thereby improving the anatomical accuracy of subsequent analyses and 3D reconstructions.

The structures used and identified in the reconstruction are as follows: the metacarpal II, III and IV bones, suspensory ligament (SL), accessory ligament of the deep digital flexor tendon (AL-DDFT), deep digital flexor tendon (DDFT), superficial digital flexor tendon (SDFT), palmar fascia with the metacarpal flexor retinaculum (MFR), and common synovial sheath (*Vag. synovialis communis mm. flexorum*, CSS); medial palmar artery and vein; and the lateral and medial palmar nerves. Surface meshes were generated using the generation surface module. Each structure was assigned a distinct color code to enhance visual differentiation and educational usability (Figure 2). The anatomical elements could be grouped, isolated, or visualized in combination, allowing interactive inspection of the topographical arrangement of the region.

Finally, the reconstructed 3D anatomical model was rendered and exported in OBJ and STL formats, making it suitable for use in external visualization platforms and a range of practical applications such as anatomy education, surgical training, virtual simulation, and 3D printing. Additionally, standardized MPEG format video files demonstrating controlled rotation, sequential layer visualization, and structure-by-structure presentation were generated to support teaching, research, and virtual museum integration.

## 3. Results

Following plastination, a total of 29 sections, each 2 mm thick, were obtained from the proximal part of the metacarpal region. Tissue loss between sections was measured at approximately 2 mm. The clear, transparent slices allowed detailed visual and microscopic study of muscles, nerves, vessels, and membranes in the proximo-palmar metacarpal area.

**Figure 3:**
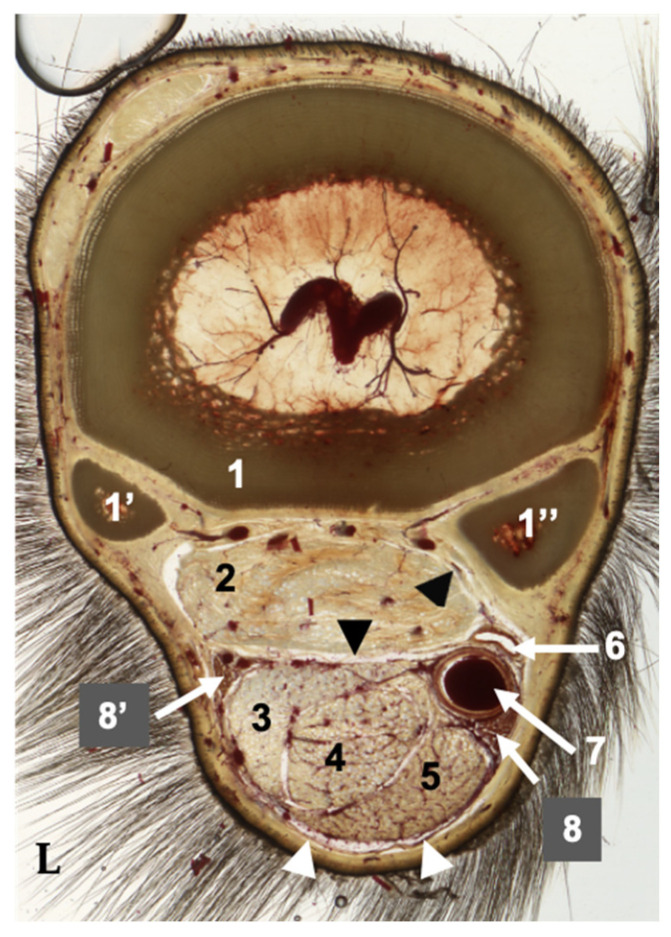
Plastinated cross-section metacarpal region with Type II AL-DDFT. 1. Metacarpal bone III; 1′. Metacarpal bone IV; 1″. Metacarpal bone II; 2. SL: Suspensory ligament; 3. Accessory ligament of the deep digital flexor tendon (AL-DDFT); 4. Deep digital flexor tendon (DDFT); 5. Superficial digital flexor tendon (SDFT); 6. Medial palmar vein; 7. Medial palmar artery; 8. Medial palmar nerve; 8′. Lateral palmar nerve; Black arrowheads: Palmar fascia with the metacarpal flexor retinaculum (MFR); White arrowheads: Common synovial sheath (CSS); L: Lateral.

Plastinated slices were evaluated from proximal to distal. The following anatomical structures were identified in full detail: the metacarpal II, III, and IV bones, DDFT, SDFT, SL, AL-DDFT, MFR, CSS, medial palmar artery and vein, and the lateral and medial palmar nerves (Figure 3). Synovial spaces between structures were also observed.

**Figure 4:**
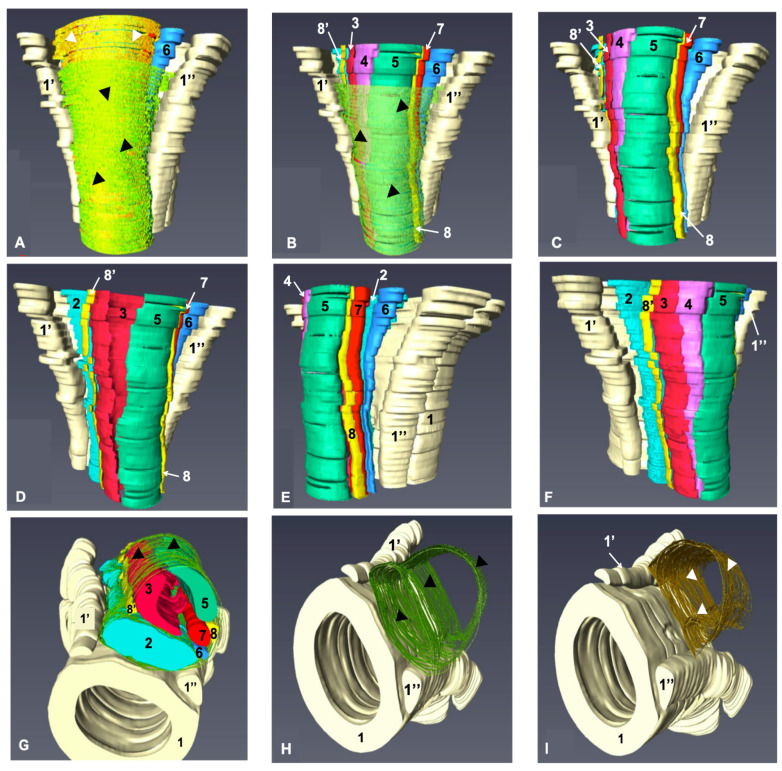
Three-dimensional rendering of the proximal palmar metacarpal region, illustrating the topographical relationships of the anatomical structures. View of the structures from palmar (**A**–**D**), palmaromedial (**E**), palmarolateral (**F**) and distopalmar (**G**–**I**). The figure illustrates the topographical relationships of anatomical structures in their native orientation. Surrounding anatomical components, including the palmar fascia and common synovial sheath, have been selectively included or excluded in each view to improve clarity of spatial configuration. 1. Metacarpal bone III; 1′. Metacarpal bone IV; 1″. Metacarpal bone II; 2. SL: Suspensory ligament; 3. Accessory ligament of the deep digital flexor tendon (AL-DDFT); 4. Deep digital flexor tendon (DDFT); 5. Superficial digital flexor tendon (SDFT); 6. Medial palmar vein; 7. Medial palmar artery; 8. Medial palmar nerve; 8′. Lateral palmar nerve; Black arrowheads: Palmar fascia with the metacarpal flexor retinaculum (MFR); White arrowheads: Common synovial sheath (CSS).

The sharp definition of structures in the plastinated slices enabled the construction of a 3D model. During the modeling process using Amira software, the following structures were reconstructed: the metacarpal II, III, and IV bones; palmar fascia with the MFR, CSS, DDFT, SDFT, SL, AL-DDFT, medial palmar artery and vein; and the lateral and medial palmar nerves (Figure 4). All these structures were color-coded; however, the palmar fascia and metacarpal flexor retinacula could not be reliably distinguished during 3D modeling because they appeared as a single, continuous connective tissue layer with no visually discernible boundaries. Therefore, for clarity and consistency in the model, both structures were assigned the same color code.

In the model, anatomical structures were grouped or displayed individually and could be freely rotated in three dimensions for detailed examination (Supplementary Video S1). Specific structures such as tendons, ligaments, nerves, and blood vessels on the palmar surface were selectively removed and re-added as needed, allowing focused analysis from different angles (Figure 4). This interactive approach made it possible to study the spatial and geometric relationships between structures, especially along the proximo-distal (or distop-roximal) direction, and better understand the spaces between them. Similarly, membranous structures, such as the palmar fascia with the MFR and the CSS, could be visualized either individually or in combination with other anatomical elements, allowing comprehensive assessment of their spatial relationships within the region (Figure 4A,B,G–I).

## 4. Discussion

A high-resolution three-dimensional reconstruction of the proximal palmar metacarpal region was achieved using E12 plastinated sections and AMIRA software, encompassing bones, tendons, ligaments, and the intricate connective tissues. Unlike previous equine anatomical models, this approach preserves the layered organization and allows precise visualization of delicate structures such as the palmar fascia with the MFR and CSS [14,15,16,17]. Minimal tissue shrinkage and the absence of deformation in the E12 plastinated sections allowed the identification of structures together with their surrounding connective tissues, supporting their accurate transfer into the digital model [2]. Accordingly, the present work provides a clearer understanding of regional anatomical relationships and establishes a foundation for the effective application of digital plastination-based three-dimensional modeling in large animal extremities [2,18,19].

The generated 3D model in this study is consistent with both the basic anatomical details and the topographical location of the structures in the plastinated sections [20,21,22]. This model can be easily positioned and rotated in 3D. The model also represents the extension of the AL-DDFT to the SDFT, and the AL-DDFT is classified as Type II [23,24]. Museums in anatomy departments increase students’ pre-dissection knowledge and preparation levels through the resources they provide, contributing significantly to their motivation to study [7,25]. When these museums are supported by technology, student access, interaction, and learning flexibility further improve [7,25]. This 3D model could be integrated into virtual learning environments, allowing users to explore, manipulate, and combine anatomical structures interactively, thus making equine anatomy more accessible to undergraduate students.

Beyond educational applications, this model may also benefit clinicians, researchers, and professionals engaged in continuous training by providing a digital resource for studying and understanding equine anatomy. Future studies should evaluate how this model enhances both educational and research platforms, particularly for users who rely on digital anatomy resources for teaching, studying, or clinical preparation.

The proximal palmar metacarpal region is among the most common sites of injury in horses [9,10]. Due to its complex anatomy, the structures in this region are challenging for students during clinical practice and also create difficulties in clinical diagnostic evaluations [9,26,27]. It is well known that changes in blood flow within the foot play a significant role in the pathogenesis of equine laminitis [28], and perineural anesthesia is an important tool in diagnosing the cause of lameness [9]. Therefore, understanding the regional anatomy is essential for diagnostic examinations, and this region has become a major focus for diagnostic and therapeutic research as well as the development of new technologies [29]. One of the major strengths of the 3D model developed in this study is its precise representation of the palmar vessels and nerves, as well as the detailed reconstruction of the AL-DDFT and the common synovial sheath. The accurate modeling of the AL-DDFT, together with the surrounding fascia and synovial structures, provides clinically relevant information for understanding disorders of the proximal metacarpus, where this ligament is frequently involved. This high level of anatomical accuracy makes the model an invaluable reference for clinicians, enhancing both diagnostic evaluations and clinical training, and provides a useful resource for future anatomical and imaging studies. Future studies should include validating the model in clinical simulations to assess its accuracy during diagnostic and surgical planning. In this study, the palmar fascia, MFR, and CSS were clearly shown in the plastinated sections, providing detailed anatomical knowledge. There has been an increase in research and applications of the palmar fascia in acupuncture and other alternative therapies [30,31]. These anatomical structures can be demonstrated by dissection, and there are limitations in digital transfer and imaging techniques. Therefore, the model provides sufficient detail to support students and clinicians in assessing the relationship of these membranes to other structures in the region, as well as to guide anatomically both invasive and non-invasive treatments.

Despite the advantages of plastination and AMIRA, some limitations remain. AMIRA, widely used for morphological and anatomical studies [32,33], is ideal for model reconstruction, but its steep learning curve prolongs modeling time [33,34]. Each plastinated section was scanned on both sides and manually segmented to compensate for low soft tissue contrast, improving accuracy, but as a common issue in digital soft tissue modeling, some artifacts still remain in the final model [33,34].

Slice thickness directly affects the geometric accuracy of the model [35,36]. Although very thin slices provide high image quality, wrinkling of tendons and ligaments was observed in slices 1 mm or thinner from E12 epoxy metacarpal blocks during the experimental phase. We believe that the primary cause of these wrinkles is the increased tissue elasticity due to the methylene chloride (MCL) used, resulting in different shrinkage behaviors of structures with different fat contents [19,37]. Therefore, increasing the slice thickness both preserved structural integrity and prevented deformations that could have occurred during modeling in this study. However, due to the increased slice thickness, stair-step artifacts were observed in the final model. In future studies, models can be created with sections obtained from acetone plastination to reduce this problem.

## 5. Conclusions

In conclusion, this study presents a 3D model created from plastinated sections that accurately depicts anatomical structures without altering their natural positions. While imaging techniques are widely used in anatomical studies, a comprehensive 3D model capable of depicting all the key structures of the equine metacarpal region in detail, including blood vessels, nerves, and connective tissues, has been lacking. The model developed in this study accurately and reliably reflects the anatomy of the region for both educational and clinical applications and can be used as a complementary tool to other imaging methods. It can be viewed from different angles, clearly demonstrating the organization and connections of the structures. It also helps identify the palmar fascia with the metacarpal flexor retinaculum (MFR) and common synovial sheath (CSS), demonstrating the sensitivity and usability of the method. The E12 plastinated sections and the 3D model complement each other and provide a strong foundation for understanding the region. The obtained results demonstrate that plastinated images can be a reliable resource for creating 3D anatomical models. The final model is a valuable tool for students, researchers, and specialists seeking to explore the anatomy of this region in depth, and it fills a significant gap in the literature. Moreover, the methodology described here could be extended to other complex and clinically relevant equine anatomical sites, such as the carpal canal, distal limb, and stifle, highlighting its potential for broader applications in both anatomical research and clinical practice.

## Data Availability

Data are contained within the article and Supplementary Materials.

## Supplementary Materials

The following supporting information can be downloaded at: https://www.mdpi.com/article/10.3390/ani16030449/s1. Video S1: Three-dimensional visualization of the model along the Z-axis illustrates the complete structures of the modeled palmar metacarpal region. Video S2: Three-dimensional visualization of the model along the X-axis illustrates the complete structures of the modeled palmar metacarpal region. Video S3: Three-dimensional visualization of the metacarpal-palmar region model showing the palmar fascia with the metacarpal flexor retinaculum (MFR) and the common synovial sheath (CSS). Video S4: Three-dimensional visualization of the model displaying the accessory ligament of the deep digital flexor tendon (AL-DDFT), deep digital flexor tendon (DDFT), superficial digital flexor tendon (SDFT), palmar fascia with MFR (MFR), and the common synovial sheath (CSS). Abbreviations with color codes: MFR: palmar fascia with the MFR; CSS: common synovial sheath; SDFT: Superficial digital flexor tendon; DDFT: deep digital flexor tendon; AL-DDFT: accessory ligament of the deep digital flexor tendon; SL: suspensory ligament; Mc II-III-IV: metacarpal bone II, III, and IV; MPA: medial palmar artery; MPV: medial palmar vein; M-L PN: medial and lateral palmar nerves.
